# Beyond coincidence: a case series on the clinical course of coinciding cluster headache and short-lasting unilateral neuralgiform headache attacks

**DOI:** 10.1097/PR9.0000000000001271

**Published:** 2025-04-28

**Authors:** Julia J. Jansen, Job Scheurink, Tijmen Balvers, Rolf Fronczek, Wim M. Mulleners

**Affiliations:** a Department of Neurology, Canisius-Wilhelmina Ziekenhuis, Nijmegen, the Netherlands; b Radboud University Medical Center, Nijmegen, the Netherlands; c Department of Epilepsy, Stichting Epilepsie Instellingen Nederland (SEIN), Heemstede, the Netherlands; d Department of Neurology, Leiden University Medical Center, Leiden, the Netherlands; e Stichting Epilepsie Instellingen Nederland (SEIN), Sleep-Wake Centre, Heemstede, the Netherlands; f The Migraine Clinic, Amsterdam, the Netherlands.

**Keywords:** Cluster headache, SUNCT, SUNA, SUNHA, Microvascular decompression, Case series

## Abstract

**Introduction:**

Limited research has been conducted on co-occurring cluster headache and short-lasting unilateral neuralgiform headache attacks (SUNHA).

**Objectives:**

This case series aims to explore whether the observed overlap of cluster headache and SUNHA reflects a coincidental co-occurrence or suggests a shared underlying mechanism while also providing insights to improve diagnosis and management of these patients.

**Methods:**

A retrospective case series of 10 patients with a diagnosis of both cluster headache and SUNHA.

**Results:**

Two patients exhibited simultaneous onset of cluster headache and SUNHA (*concurrent cluster-SUNHA*), whereas the remaining 8 had *consecutive cluster-SUNHA*. Overlapping localization of pain and autonomic symptoms were observed in 9 of 10 cases. Magnetic resonance imaging was performed in 8 patients, revealing an ipsilateral trigeminal neurovascular conflict in half of them. Four patients underwent microvascular decompression, which exhibited varying efficacy on both cluster headache and SUNHA.

**Conclusion:**

This study constitutes the most extensive description to date of individuals diagnosed with both cluster headache and SUNHA. The observed co-occurrence of these headache types could suggest a pathophysiological relationship between the 2, which might be explained by a bidirectional interaction between posterior hypothalamic function and trigeminal root demyelination. The role of a trigeminal neurovascular conflict seems likely in this context, with dedicated magnetic resonance imaging being the optimal method for its identification. The potential role of microvascular decompression as a treatment in cluster-SUNHA cases where such a conflict is identified warrants further investigation.

## Abstract 2

A case series of 10 patients with co-occurring cluster headache and short-lasting unilateral neuralgiform headache attacks, addressing clinical management, including microvascular decompression, and exploring a potential shared pathophysiological link.

## 1. Introduction

Trigeminal autonomic cephalalgias (TACs) are characterised by unilateral attacks and ipsilateral cranial autonomic features.^1,7^ Cluster headache (CH), the most common TAC, presents as severe attacks, typically localized in the first trigeminal nerve division, lasting 15 to 180 minutes, often accompanied by restlessness or agitation. In episodic CH, attacks occur in bouts with remission periods, whereas chronic CH lacks attack-free intervals of at least 3 months.^14^ Short-lasting unilateral neuralgiform headache attacks (SUNHA) last 1 to 600 seconds and includes short-lasting unilateral neuralgiform headache attacks with conjunctival injection and tearing (SUNCT) and short-lasting unilateral neuralgiform headache attacks with cranial autonomic symptoms (SUNA).^23^ Short-lasting unilateral neuralgiform headache attack subtypes are hypothesized to be diverse manifestations of the same disorder.^10^ Co-occurrence of TAC subtypes is well-known and typically involves overlap of CH and paroxysmal hemicrania.^20^ Cluster headache–short-lasting unilateral neuralgiform headache attack co-occurrence has been rarely reported.^16,20^ Because we encountered several patients with CH-SUNHA, it raised the question whether they are truly separate biological entities, prompting an investigation into their clinical characteristics, temporal relationship, and neuroimaging findings. This study aims to explore whether these characteristics may shed light on a possibly shared underlying mechanism while also providing insights to improve diagnosis and management of these patients. Moreover, pathophysiological implication will be reviewed and discussed.

## 2. Methods

A retrospective study identified subjects with SUNHA and CH across 4 Dutch hospitals. Data collected from medical records included demographics, pain characteristics (with emphasis on localization and laterality), temporal relationships, intoxications, comorbidities, neurological examination findings and current and past treatments. MRIs were evaluated by a headache specialist and neuroradiologist, using the classification by Sindou et al.^18^ All data were anonymized and clinical characteristics were reassessed to confirm diagnoses according to the International Classification of Headache Disorders, 3rd edition.^7^ Data were analysed using descriptive statistics. The institutional review board waived formal ethical approval. Written informed consent was obtained.

## 3. Results

### 3.1. Participants

Ten patients were included. The mean age of onset was 44 (range: 16–72) years for CH and 48 (range: 17–75) years for SUNHA. Table 1 summarizes the characteristics.

**Table 1:** Characteristics of patients with co-existent cluster headache and short-lasting unilateral neuralgiform headache attacks.

Patient	1	2	3	4	5	6	7	8	9	10
SUNHA										
Subtype	SUNCT	SUNA	SUNA	SUNCT	SUNCT	SUNCT	SUNCT	SUNA	SUNCT	SUNCT
Gender	F	M	M	M	M	M	M	M	F	F
Age of onset (y)	49	36	71	75	17	60	42	61	33	34
Untreated attack duration (s)	120–180	Unknown	1	120–600	1–180	1	1–120	1–180	1–3	2–120
Attack frequency (per day)	Unknown	20	10–50	8–10	30	6–7	40–100	100	60	30
Autonomic features	Tearing, CI, ptosis, rhinorrhoea	Tearing, NC	Tearing	Tearing, CI	Tearing, CI	Tearing, CI, NC	Tearing, CI, ES	Tearing and rhinorrhoea	Tearing, CI, ES, ptosis, rhinorrhoea	Tearing, CI
Quality of pain	Unknown	Stabbing	Stabbing	Stabbing	Stabbing	Stabbing or saw-tooth pattern	Stabbing	Stabbing	Stabbing	Stabbing
Triggered	Unknown	Unknown	Sometimes	Sometimes	Sometimes	No	Sometimes	No	Sometimes	No
(Partial) Effective preventive treatment	—	—	LTG 200	CBZ 600	LTG 700 + OXC 600 (partial)	LTG 300 (partial)	CBZ 1200 + LTG 300 (partial)	—	Botulinum toxin	OXC 300, PGB, GBP (partial effects)
Ineffective preventive treatments	LTG 125	LTG 150, CBZ 400	—	IND	CBZ 800	CBZ 300	—	LTG 600 + OXC 300	LTG	—
Outcome	Resistant	Lost to follow-up	Remission	Remission	Temporary remission	Resistant	Remission	Resistant	Resistant	Near remission
Cluster headache										
Type	Chronic	Chronic	Episodic	Episodic	Chronic	Chronic	Episodic	Episodic	Chronic	Chronic
Age of onset of CH (y)	48	34	57	72	16	44	41	61	33	36
Untreated attack duration (min)	90	120	30–120	120	30–75	30–60	45–90	150–180	120–180	30–90
Attack frequency (per day)	8	4–8	8	Multiple times daily to biweekly	<1	8–10	3	<1	2	4
Accompanying symptoms	R/A, tearing, CI, ptosis, rhinorrhoea	R/A, tearing, rhinorrhoea, NC, ptosis, miosis, FS	Rhinorrhoea, tearing, CI	CI, tearing	R/A, ptosis, ES, CI	CI, tearing, rhinorrhoea	CI, tearing	R/A, tearing, rhinorrhoea, NC	Tearing, CI, ES, FS, rhinorrhoea	R/A, CI, tearing, NC, ptosis
Effective attack treatment	None	O_2_, SUC, SUN	SUC, SUN	O_2_, SUN	SUC	O_2_, SUN, fentanyl i.n.	None	None	O_2_, SUN	O_2_
(Partial) Effective preventive treatment	—	—	VER 120, GON	VER	VER, PRE	GON 80	VER 400, PRE 50, GON 40	VER 360, GON 80	TOP, ONS, vitamin D	VER 320, TPM 30 (partial), GBP, PRB
Ineffective preventive treatments	GON 80, PRE, PRF, VER 720, LIT 800	TOP 75	—	TOP	—	VER, LIT	—	—	VER, LIT, GON, GBP	GON, PRF
Outcome	Resistant	Lost to follow-up	Remission	Remission	Remission	Remission	Remission	Continuing relapses	Reduction	Reduction
Ipsilateral NVC	No	Unknown	No	Yes	Yes	Unknown	Yes	Yes	No	No
MVD performed*	No	No	No	No	Yes	No	Yes	Yes	No	Yes
Other headaches	—	Shadow pain in CH region	—	TN preceding SUNHA in same region	—	Shadow pain forehead bilateral	Shadow pain in CH region; 1st and 3rd *contra*lateral branch TN	—	—	TN in SUNHA region

*Clinical characteristics:* CBZ, carbamazepine; CI, conjunctival injection; ES, eyelid swelling; FS, facial sweating; *Medication (daily dose in mg when applicable and available);* GBP, gabapentin; GON, greater occipital nerve block with methylprednisolone; IND, indomethacin; LIT, lithium; LTG, lamotrigine; NC, nasal congestion; O_2_, oxygen 100%; OXC, oxcarbazepine; PGB, pregabalin; PRE, prednisolone; PRF, sphenopalatine ganglion block; R/A, restlessness or agitation; SUC, sumatriptan subcutaneous 6 mg; SUN, sumatriptan 20 mg intranasal; TPM, topiramate; VER, verapamil.

*Other:* MVD, microvascular decompression; NVC, neurovascular conflict; ONS, occipital nerve stimulation; TN, trigeminal neuralgia.

* See Table 2.

### 3.2. Time course of cluster headache and short-lasting unilateral neuralgiform headache attacks

**Figure 1.**
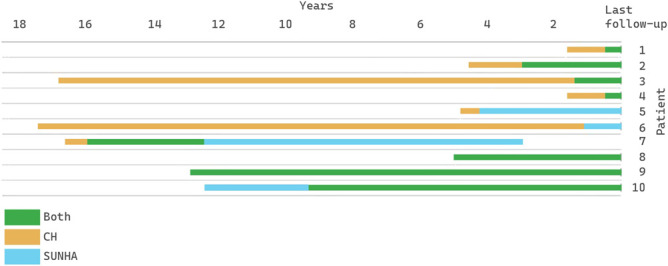
Temporal relationship between CH and SUNHA from diagnosis to last follow-up. CH, cluster headache; SUNHA, short-lasting unilateral neuralgiform headache attacks.

Of 10 subjects, 6 had chronic and 4 episodic CH. Seven subjects initially presented with CH (group 1) and 1 with SUNHA (group 2). In 2 subjects, CH and SUNHA started simultaneously (group 3) (Fig. 1).

In group 1, the time to onset of SUNHA ranged from 0.5 to 16 years, averaging 5.3 years. Five patients developed SUNHA while CH persisted. Two converted into SUNHA, which occurred in close temporal relation to a methylprednisolone/lidocaine greater occipital nerve block in 1. Spontaneous resolution of headaches occurred in 1 subject: CH resolved after 4.1 years, SUNHA after an additional 9.5 years.

In 1 subject in group 2, SUNHA preceded CH by 3 years.

### 3.3. Pain and autonomic symptoms

All patients initially had ipsilateral attacks, with pain and autonomic symptoms during CH and SUNHA on the same side. However, over time, 2 subjects experienced alternating attack sides. One predominantly experienced right-sided CH and SUNHA, with occasional shifts. The other reported side shifts for CH only.

**Figure 2.**
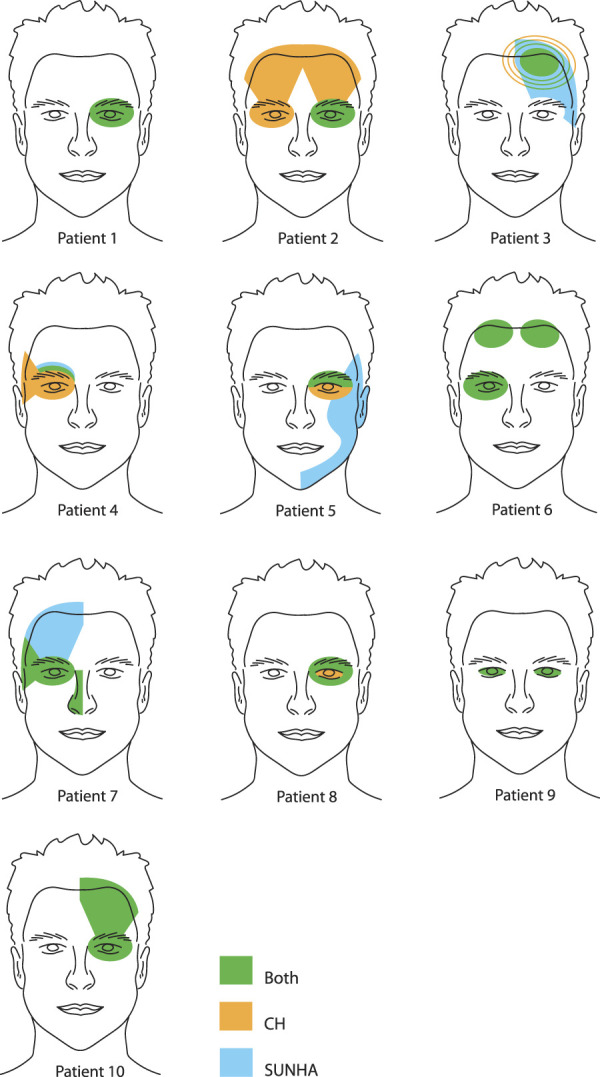
Pain localization of CH, SUNHA, or the combination of both. In patient 2 and 6, side-shifts occurred. CH, cluster headache; SUNHA, short-lasting unilateral neuralgiform headache attacks.

All patients reported pain in the peri-orbital, supra-orbital, or temporal region with CH attacks. Nine patients had SUNHA in that same cranial region, whereas in 1 SUNHA, pain extended into V2 and V3 (Fig. 2). Nine patients reported similar autonomic symptoms during CH and SUNHA (Table 1).

### 3.4. Other headache disorders

Three patients (30%) had dual diagnoses of trigeminal neuralgia (TN) and SUNHA, based on their temporally separated occurrence and prominent cranial autonomic symptoms in the latter. In 2 cases, the localization of TN differed from SUNHA. Trigeminal neuralgia and short-lasting unilateral neuralgiform headache attacks were elicited by typical triggers in 2. Three patients reported concomitant continuous “shadow pain” in the same region as CH.

### 3.5. Neurological assessment

Neurological examination revealed abnormalities in 2 patients. In 1 patient, mild slowness of speech and motor task execution was observed, cognitive and neurological examination as well as MRI of the brain parenchyma were otherwise unremarkable. One demonstrated a unilateral extensor response, alongside vascular white matter lesions on neuroimaging.

### 3.6. Radiological assessment

Dedicated imaging of the trigeminal area was available in 8 patients, and all were reviewed by a headache specialist and neuroradiologist. Four patients (50%) had an ipsilateral neurovascular conflict (NVC). In a previously published and reclassified case (patient 10),^4^ the suspected petrosal vein compression was not confirmed in our MRI review procedure. Not all MRIs could be assessed for the presence of contralateral NVC. Table 2 lists imaging protocols and review. One subject had a pituitary micro adenoma.

**Table 2:** Magnetic resonance imaging characteristics of patients with co-existent cluster headache and short-lasting unilateral neuralgiform headache attacks and (if applicable) peroperative findings and postoperative effect.

Patient	Dedicated MRI	Slice thickness	1D/3D	TOF	Contrast	NVC laterality to SUNHA		Ipsilateral NVC specifics		MVD	Peroperative findings	Effect of MVD (when applicable)
						Ipsilateral	Contralateral	Arterial/venous	Grade			
1	Yes	0.5 mm	3D	No	No	No	Na	—	—	No	—	
3	Yes	0.5 mm	1D	No	Yes	No	Na	—	—	No	—	
4	Yes	0.5 mm	3D CISS	No	No	Yes	No	Arterial (SCA)	2–3	No	—	
5	Yes	0.5 mm	1D CISS	No	No	Yes	No	Venous	1	Yes	Arachnoid adhesions constricting CN V, with clear vein-induced indentation. No arterial conflict	CH: in remission year before MVD SUNHA: complete remission of attacks post-MVD, allowing discontinuation of LTG. Recurrence 5 mo later. No new NVC. Medical treatment resumed and modified on symptom fluctuations during 3-y FU
7	Yes	0.7 mm	1D CISS	No	No	Yes	Yes	Arterial (SCA)	3	Yes	Compression of CN V by SCA and superior petrosal vein	CH: no new bout 6 y before MVD. SUNHA: remission on treated side (5 y FU)
8	Yes	0.7 mm	1D	No	No	Yes	No	Arterial (SCA) and venous	2	Yes	CN V indentation, distortion and gray discoloration from vascular structures (AICA, SCA). Plus venous contact	CH and SUNHA: no effect of MVD on either condition; medical treatment continued until last FU (1.5 y post-MVD)
9	Yes	0.5 mm	1D	No	No	No	Na	—	—	No	Na	
10	Yes	0.5 mm	1D	No	Yes	No*	Na	—	—	Yes	Compression of the trigeminal nerve by a petrosal vein	CH: only temporary reduction in frequency and intensity of attacks (duration unknown). Still treated for CH 7 y later SUNHA: near remission of attacks up to 7 y FU

* At the initial MRI assessment in the report, no NVC was seen. However, when other treatments failed, MVD was performed. On revisiting the MRI, compression by the petrosal vein was suspected. Nevertheless, in this case series, both the headache specialist and the neuroradiologist found no evidence of NVC upon MRI reassessment.

AICA, anterior inferior cerebellar artery; CN V, trigeminal nerve; FU, follow-up; LTG, lamotrigine; MVD, microvascular decompression; Na, not assessed; NVC, neurovascular conflict; SCA, superior cerebellar artery; TN, trigeminal neuralgia; TOF, time of flight angiography.

### 3.7. Treatment and responses

Table 1 lists medical treatment and responses. Table 2 provides details of follow-up after MVD (when applicable).

## 4. Discussion

Our study adds a substantial number of cases describing several presentations for co-occurring CH and SUNHA. The male-to-female ratio in this series (2.3:1) was comparable with European and North American CH populations (2–3:1).^21^ Regarding the temporal course of CH and SUNHA, 8 patients had *consecutive* cluster-SUNHA, whereas *concurrent* cluster-SUNHA (defined as simultaneous onset of both) occurred in just 2 patients. Seven consecutive cluster-SUNHAs started with CH, with 4 evolving into co-occurring CH and SUNHA, making this the predominant type. Headache type interval was generally short, averaging 4 years with outliers at 15 and 16 years.

We believe that our findings argue in favour of a pathophysiologic association between CH and SUNHA. Firstly, in 9 cases, both occurred on the same side and area, with similar autonomic symptoms. Secondly, the generally short interval between headache types suggests noncoincidence. Thirdly, reported cases exceed expected chance occurrences. Considering the prevalence of CH and SUNHA, random association would result in 1.19 patients with both disorders in the Netherlands (18.028.211 inhabitants), half not being ipsilateral.^14,19,23^ The actual number is probably higher as misdiagnosis of either entity, the retrospective study design and nonsystematic case selection, may lead to prevalence underestimation. Finally, the temporary effect of MVD on CH in 1 patient was interesting because it could suggest NVC influencing the pathophysiology of CH. However, the circannual rhythm of CH should be considered when evaluating MVD effects, as only 2 subjects were treated while in-bout, precluding efficacy inferences. Nevertheless, MVD has been recommended for ipsilateral NVC in SUNHA.^17^ Although this is a retrospective study, evidence for the (long-term) efficacy of MVD may not be inferred.

Although the pathophysiology of neither CH nor SUNHA is completely understood, hypothalamic dysfunction is implicated in both. The hypothalamus modulates brainstem nociceptive and autonomic responses and its dysfunction is thought to cause disinhibition of the hypothalamic-trigeminal pathway and trigeminal-autonomic reflex activation.^2,9,10,12,14^ However, while nociceptive signalling in CH is primarily dependent on trigeminovascular activation, some consider SUNHA as a trigeminal neuralgic disorder at the root entry zone, similar to TN.^9,10^ Unlike TN, central disinhibition of the trigeminal nucleus caudalis (TNC) may explain the lack of refractory periods in SUNHA.^8,9^ This notion, however, does not entirely explain how CH and SUNHA may co-occur. Interestingly, debate is open about the mechanisms in the resembling cluster-tic overlap syndrome. Some adhere to the view that these diagnoses are mere comorbidities.^5,22^ Others propose a disturbance of hypothalamic neurotransmitter regulation, predisposing to pathological activation of the trigemino-hypothalamic pathway, and hence of neurons in the TNC.^13^

We hypothesize that in CH, failing hypothalamic TNC modulation may not only contribute to dysfunctional neurotransmission in the trigeminovascular system^15^ but also lead to release of hitherto subclinical trigeminal neuralgic signalling and hence a SUNHA phenotype. As NVC was observed in half of our imaged subjects and its high prevalence in SUNHA has been reported before,^11^ we believe that a significant mechanistic contribution of a peripheral trigeminal component is likely.^24^ This hypothesis gains credibility as CH occurred before or alongside SUNHA in most cases. Conversely, trigeminal root demyelination could alter posterior hypothalamic function, rendering SUNHA patients more susceptible to CH. However, as CH preceded SUNHA in most and the evidence supporting MVD in CH is limited, the latter scenario is less likely. Nevertheless, a bidirectional interaction could be the differentiating factor between SUNHA and cluster-SUNHA.

Notably, 2 patients reported side-shifts, limited to CH attacks in one, whereas both headache types shifted in another. This phenomenon has been described in both CH and SUNHA.^2,3,6,15^ Furthermore, we observed 3 patients with both TN and SUNHA. This may reflect misclassification because of the retrospective description of symptoms, challenging unequivocal attribution to diagnostic entities displaying subtle differences in presence of a refractory period, typical pain distribution, likelihood of NVC with morphological changes and severity of autonomic features.^10,24^ However, in our view, it is consistent with the notion that SUNHA and TN could represent variants of the same disorder.

This study offers the most extensive description to date of coinciding CH and SUNHA. presenting as either concurrent or consecutive cluster-SUNHA. It provides valuable insights for the recognition and clinical management of these patients. In addition, it could enhance future trial designs by reducing clinical and radiological heterogeneity. The findings support a hypothesis regarding a potential pathophysiological connection between these headache types. Co-occurring CH and SUNHA may be attributable to release of trigeminal neurovascular signalling caused by a lack of hypothalamic control of TNC processing.

## Disclosures

R.F. has received research support from the Netherlands Brain Foundation and ZonMw and did paid advisory work for Lilly, Takeda, Bioprojet, Jazz Pharma, Novartis, and Teva. W.M. has received research support from ZonMw. These affiliations have not influenced the content of this article.

## References

[1] Alberca R Ochoa JJ. Cluster tic syndrome. Neurology 1994;44:996–9. 8208435 10.1212/wnl.44.6.996

[2] Benoliel R Sharav Y Haviv Y Almoznino G. Tic, triggering, and tearing: from CTN to SUNHA. Headache 2017;57:997–1009. 28188632 10.1111/head.13040

[3] Brandt RB Naber WC Ouwehand RLH Haan J Ferrari MD Fronczek R. Transient side shift of cluster headache attacks after unilateral greater occipital nerve injection. Headache 2023;63:1193–7. 37358558 10.1111/head.14587

[4] de Coo I van Dijk JM Metzemaekers JD Haan J. A case report about cluster-tic syndrome due to venous compression of the trigeminal nerve. Headache 2017;57:654–7. 27925184 10.1111/head.12990

[5] Goadsby PJ Lipton RB. Paroxysmal hemicrania-tic syndrome. Headache 2001;41:608–9. 11437905 10.1046/j.1526-4610.2001.041006608.x

[6] Groenke BR Daline IH Nixdorf DR. SUNCT/SUNA: case series presenting in an orofacial pain clinic. Cephalalgia 2021;41:665–76. 33269943 10.1177/0333102420977292

[7] Headache classification committee of the International Headache Society (IHS) the international classification of headache disorders, 3rd edition. Cephalalgia 2018;38:1–211. 10.1177/0333102417738202 29368949

[8] Hoffmann J Baca SM Akerman S. Neurovascular mechanisms of migraine and cluster headache. J Cereb Blood Flow Metab 2019;39:573–94. 28948863 10.1177/0271678X17733655 PMC6446418

[9] Kang MK Cho SJ. SUNCT, SUNA and short-lasting unilateral neuralgiform headache attacks: debates and an update. Cephalalgia 2024;44:3331024241232256. 38415675 10.1177/03331024241232256

[10] Lambru G Matharu MS. SUNCT, SUNA and trigeminal neuralgia: different disorders or variants of the same disorder? Curr Opin Neurol 2014;27:325–31. 24792341 10.1097/WCO.0000000000000090

[11] Lambru G Rantell K O'Connor E Levy A Davagnanam I Zrinzo L Matharu M. Trigeminal neurovascular contact in SUNCT and SUNA: a cross-sectional magnetic resonance study. Brain 2020;143:3619–28. 33301567 10.1093/brain/awaa331 PMC7807031

[12] Leone M Bussone G. Pathophysiology of trigeminal autonomic cephalalgias. Lancet Neurol 2009;8:755–64. 19608101 10.1016/S1474-4422(09)70133-4

[13] Leone M Mea E Genco S Bussone G. Coexistence of TACS and trigeminal neuralgia: pathophysiological conjectures. Headache 2006;46:1565–70. 17115989 10.1111/j.1526-4610.2006.00537.x

[14] May A Schwedt TJ Magis D Pozo-Rosich P Evers S Wang SJ. Cluster headache. Nat Rev Dis Primers 2018;4:18006. 29493566 10.1038/nrdp.2018.6

[15] Meyer EL Laurell K Artto V Bendtsen L Linde M Kallela M Tronvik E Zwart JA Jensen RM Hagen K. Lateralization in cluster headache: a Nordic Multicenter Study. J Headache Pain 2009;10:259–63. 19495933 10.1007/s10194-009-0129-z PMC3451747

[16] Russo A Silvestro M Tessitore A Tedeschi G. The “Cluster-SUNCT syndrome”: the lumper-splitter problem. Pain Med 2019;20:421–3. 30107598 10.1093/pm/pny161

[17] Sebastian S Schweitzer D Tan L Broadley SA. Role of trigeminal microvascular decompression in the treatment of SUNCT and SUNA. Curr Pain Headache Rep 2013;17:332. 23564233 10.1007/s11916-013-0332-0

[18] Sindou M Leston J Decullier E Chapuis F. Microvascular decompression for primary trigeminal neuralgia: long-term effectiveness and prognostic factors in a series of 362 consecutive patients with clear-cut neurovascular conflicts who underwent pure decompression. J Neurosurg 2007;107:1144–53. 18077952 10.3171/JNS-07/12/1144

[19] Statistics Netherlands. Pupulation counter Netherlands. Available at: https://www.cbs.nl/en-gb/visualisations/dashboard-population/population-counter. Accessed November 11, 2024.

[20] Totzeck A Diener H-C Gaul C. Concomitant occurrence of different trigeminal autonomic cephalalgias: a case series and review of the literature. Cephalalgia 2014;34:231–5. 24065715 10.1177/0333102413506127

[21] Wei DY Yuan Ong JJ Goadsby PJ. Cluster headache: epidemiology, pathophysiology, clinical features, and diagnosis. Ann Ind Acad Neurol 2018;21(suppl 1):S3–8. 10.4103/aian.AIAN_349_17 PMC5909131 29720812

[22] Wilbrink LA Weller CM Cheung C Haan J Ferrari MD. Cluster-tic syndrome: a cross-sectional study of cluster headache patients. Headache 2013;53:1334–40. 23808839 10.1111/head.12161

[23] Williams MH Broadley SA. SUNCT and SUNA: clinical features and medical treatment. J Clin Neurosci 2008;15:526–34. 18325769 10.1016/j.jocn.2006.09.006

[24] Wöber C. Tics in TACs: a step into an avalanche? Systematic literature review and conclusions. Headache 2017;57:1635–47. 28542727 10.1111/head.13099

